# Lactoferrin’s Anti-Cancer Properties: Safety, Selectivity, and Wide Range of Action

**DOI:** 10.3390/biom10030456

**Published:** 2020-03-15

**Authors:** Antimo Cutone, Luigi Rosa, Giusi Ianiro, Maria Stefania Lepanto, Maria Carmela Bonaccorsi di Patti, Piera Valenti, Giovanni Musci

**Affiliations:** 1 Department of Biosciences and Territory, University of Molise, 86090 Pesche, Italy; g.ianiro@studenti.unimol.it; 2 Department of Public Health and Infectious Diseases, University of Rome La Sapienza, 00185 Rome, Italy; luigi.rosa@uniroma1.it (L.R.); mariastefania.lepanto@uniroma1.it (M.S.L.); piera.valenti@uniroma1.it (P.V.); 3 Department of Biochemical Sciences, Sapienza University of Rome, 00185 Rome, Italy; mariacarmela.bonaccorsi@uniroma1.it

**Keywords:** lactoferrin, cancer, lactoferrin bioavailability, tumor proliferation, apoptosis, epithelial to mesenchymal transition, metastasis, cancer targeting

## Abstract

Despite recent advances in cancer therapy, current treatments, including radiotherapy, chemotherapy, and immunotherapy, although beneficial, present attendant side effects and long-term sequelae, usually more or less affecting quality of life of the patients. Indeed, except for most of the immunotherapeutic agents, the complete lack of selectivity between normal and cancer cells for radio- and chemotherapy can make them potential antagonists of the host anti-cancer self-defense over time. Recently, the use of nutraceuticals as natural compounds corroborating anti-cancer standard therapy is emerging as a promising tool for their relative abundance, bioavailability, safety, low-cost effectiveness, and immuno-compatibility with the host. In this review, we outlined the anti-cancer properties of Lactoferrin (Lf), an iron-binding glycoprotein of the innate immune defense. Lf shows high bioavailability after oral administration, high selectivity toward cancer cells, and a wide range of molecular targets controlling tumor proliferation, survival, migration, invasion, and metastasization. Of note, Lf is able to promote or inhibit cell proliferation and migration depending on whether it acts upon normal or cancerous cells, respectively. Importantly, Lf administration is highly tolerated and does not present significant adverse effects. Moreover, Lf can prevent development or inhibit cancer growth by boosting adaptive immune response. Finally, Lf was recently found to be an ideal carrier for chemotherapeutics, even for the treatment of brain tumors due to its ability to cross the blood–brain barrier, thus globally appearing as a promising tool for cancer prevention and treatment, especially in combination therapies.

## 1. Lactoferrin: A Brief Overview

It’s just over 80 years since the glycoprotein Lactoferrin (Lf) was first discovered in bovine milk [1] and later purified as an iron-containing red protein from human milk [2]. This glycoprotein is present in milk from different species such as cow, pig, mouse, horse, rabbit, and dog and its production is species- and lactation stage-dependent [3,4]. Notably, concentration of Lf in human milk is the highest among the different species, with the maximum peak in colostrum (6.7 g/L) and lower concentrations in transitional (3.7 g/L) and mature milk (2.6 g/L) [4]. Despite its name, Lf was subsequently found to be present also in other biological fluids including saliva, tears, mucus, seminal fluid, bronchial secretions, and in secondary granules of neutrophils [5]. Lf is functionally and structurally similar to serum transferrin with 60% sequence identity [6]. As a matter of fact, this glycoprotein, also known as lacto-transferrin, is classified as a member of the Transferrin (Tf) family, in addition to melano-, ovo-, and serum-Tf (sTf) [7]. Transferrins are a superfamily of iron-binding proteins constituted by a single polypeptide chain of 650–700 residues with a two-fold internal repeat derived from an earlier gene duplication event, which gives rise to an N-lobe and a C-lobe. The two homologous lobes share about 40% sequence identity and each of them can reversibly bind a ferric ion [8]. Both Lf and sTf have high affinities for Fe^3+^ (Kd = 10^-20^ M, [9]) due to a highly conserved set of ligands for the ferric ion [10,11]. However, Lf and sTf differ in some physicochemical properties, in particular Lf has high iron binding stability at low pH, whereas sTf releases iron under such conditions [6]. This different feature reflects distinct functions of the considered proteins: indeed, sTf acts as a cargo for iron transport not only into cells [12] but also in the blood, a district that it is usually characterized by pH values in a narrow neutral range (7.2–7.4) [13], while Lf often exerts its role at inflamed and infected sites, where pH can reach acidic values. Indeed, Lf shares its anti-microbial, antifungal, antiviral, and anti-parasitic activities with ovo-Tf [14,15], whereas it possesses unique features as an anti-inflammatory, immunomodulatory, and anti-cancer molecule [16,17,18]. Moreover, it is emerging as a fundamental regulator of cellular and systemic iron homeostasis [19,20]. All the activities ascribed to Lf can be dependent or independent of its iron-binding ability.

**Figure 1:**
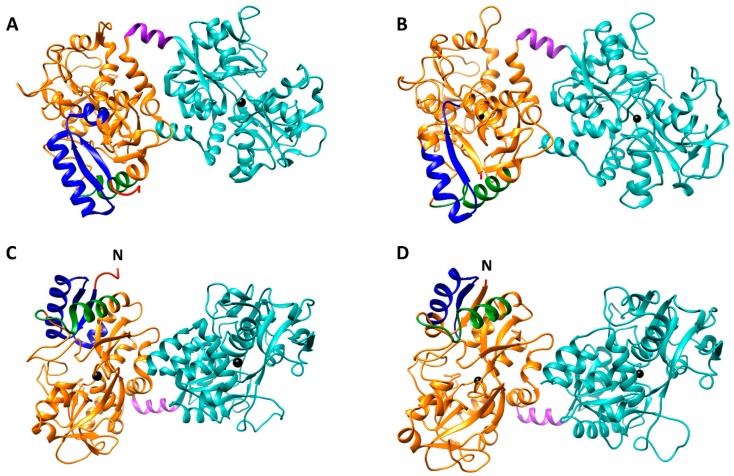
Side (**A**,**B**) and top (**C**,**D**) views of the crystal structures of diferric human lactoferrin (hLf) (**A**,**C**) (PDB code = 1B0L) and bovine lactoferrin (bLf) (**B**,**D**) (PDB code = 1BLF). The N-lobe is highlighted in orange and the C-lobe in cyan, the connecting α-helix (aa. 334–344 for both glycoproteins) in violet, and the ferric irons are depicted as black spheres. The lactoferricin (aa. 1–47 in hLf and 17–41 in bLf) and lactoferrampin (aa. 269–285 in hLf and 268–284 in bLf) regions are highlighted in blue and green, respectively. N-terminal regions, able to interact with different glycosaminoglycans on cancer cells, are depicted in red and correspond to the first five amino acids (G^1^RRRR^5^) for hLf, while in bLf only Asn in position 5 (N^5^) is shown, as the first four amino acids (A^1^PRK) are absent in the crystal structure. As evident from the top view, the cationic lactoferricin, lactoferrampin and the N-terminal region are packed close to each other and constitute a basic domain able to interact with prokaryotic and eukaryotic cell surface receptors, thus exerting multifaceted activities.

The Lf primary structure has been characterized in multiple species [9]. Human Lf (hLf) shows high similarity with other Lfs isolated from bovine (bLf), horse, and buffalo [21,22,23]. It is an 80 kDa single polypeptide chain of 691 amino acids, with the N- (residues 1–333) and C- (residues 345–691) lobes connected by a three-turn-helix peptide (residues 334–344) (Figure 1) [24]. Ferric ligands are constituted by two tyrosines (Y92 and Y192 for N-lobe and Y433 and Y526 for the C-lobe), a histidine (H253 for N-lobe and H595 for C-lobe) and an aspartic acid (D60 for the N-lobe and D395 for the C-lobe), that, along with two oxygens from a CO_3_^2-^ ion, form a binding site with an octahedral geometry [9]. Lf is a cationic protein (pI ca. 9), rich in basic amino acids especially in the N-lobe, which presents two peptide sequences, namely lactoferricin (Lfcin, aa. 1–47 in hLf and 17–41 in bLf) and lactoferrampin (Lfampin, aa. 269–285 in hLf and 268–284 in bLf), which have been described to possess their own biological functions (Figure 1) [25,26]. Both peptides can be generated by Lf tryptic digestion after oral ingestion, thus suggesting their physiological implication in gut homeostasis. Indeed, Lfcin and Lfampin are endowed with potent anti-microbial [26,27], anti-fungal [28], anti-viral [29], anti-inflammatory [30], and anti-cancer properties [31]. Most of the functions ascribed to these peptides are due to their high positive charge, which enables them to interact with negatively charged surface of both prokaryotic and eukaryotic cells, thus altering the permeability of membranes and, in case of pathogens or cancer cells, inducing cell lysis and death [31,32].

Depending on its iron content, Lf can assume two opposite conformational states, the open iron-free form (apo-Lf) and the closed iron-binding form (holo-Lf) [33]. The native form of Lf, produced and secreted in physiological conditions, has an iron saturation rate between 10% and 20%, thus leading to the prevalence of apo- and monoferric Lf and a very low content of diferric Lf. On the other hand, in inflammation and/or infection sites, characterized by high levels of free iron, the holo-form is mostly present. Interestingly, the iron content influences the physico-chemical properties of Lf [34]. First of all, holo-Lf is more stable than the unsaturated form, with higher thermal stability and a significant resistance to proteolytic digestion [34]. Some of the functions exerted by Lf can also be affected to its iron-binding status, as it is able to scavenge free iron in fluids and inflamed and/or infected sites, suppressing free radical-mediated damage and decreasing the availability of the metal to pathogens and cancer cells. In addition, many studies have shown that, depending on the iron-saturation rate, Lf can exert dissimilar functions by activating specific signaling pathways [35,36]. It is, therefore, of the utmost importance to consider the iron saturation rate when carrying out in vitro and in vivo experiments.

Lf biological function is also influenced by glycosylation, the most common protein post-translational modification affecting protein folding, immunogenicity, protein solubility, and resistance to proteolysis. Glycosylation is a species-specific and tissue-specific modification, e.g., hLf and recombinant human Lf (rhLf) contains two major glycosylation sites, Asn 138 and Asn 479 [37,38], but different N-glycan patterns [39]. Indeed, rLf expressed in cow has a lower content of sialic acid and fucose, showing high mannose-, hybrid-, and complex-type structures, while N-glycans from hLf are comprised entirely of highly branched, highly sialylated, and highly fucosylated complex-type structures [40]. Bovine, caprine and ovine Lfs present five potential glycosylation sites (Asn233, 281, 368, 476, and 545) compared to only one in murine Lf (Asn 476) [41]. However, in bLf only four sites are invariably glycated (Asn233, Asn368, Asn476, and Asn545) [21], whereas Asn281 can selectively undergo glycosylation, giving rise in this case to a higher molecular mass bLf (84kDa vs. 81kDa) [42].

Despite the structural differences reported above, bLf has been classified as and hLf bioequivalent by virtue of high sequence homology and function sharing and, in addition, it is classified as a “generally recognized as safe” (GRAS) substance by the USA Food and Drug Administration. Therefore, the majority of the in vitro [43,44] and in vivo studies [45,46], as well as clinical trials [47,48] have been conducted by using commercial bLf rather than the highly costing rhLf. Furthermore, bLf is currently used to supplement food products and cosmetics, to inhibit oxidation due to its iron-binding capacity, and in infant formulas to improve the immune response in newborn. However, differences in the primary source of bLf, and in industrial procedures, influencing bLf integrity and purity, have shown great impact on the efficacy of commercial bLf preparations, especially in vivo, where bLf bioavailability can crucially influence the success or the failure of its action.

## 2. Lactoferrin Bioavailability: Absorption and Body Delivery

Lf bioavailability represents a bizarre biological issue, since mammals are differently exposed to the action of endogenous Lf depending on age, ranging from its high abundance during infancy, through breast feeding, to low availability during development and aging. Indeed, excluding Lf ingestion in homologous or heterologous form from milk, normal diet remains a poor source of exogenous Lf, also depending on individual habits, while systemic Lf is exclusively guaranteed by neutrophils degranulation in infection and inflammatory states. In healthy condition, Lf concentration in blood is rather variable yet always relatively low, varying from 0.02 to 1.52 μg/mL, whereas it increases during infection, inflammation, excessive intake of iron, or tumor growth [49]. However, most of the in vitro studies on the multi-functional activities of this protein, including the anti-inflammatory and anti-cancer ones, showed that higher concentrations need to be used for Lf to be effective, therefore, exogenous Lf must be administered in vivo to overcome this drawback. Several tests in both animals and humans have proven its safety and tolerability, even at high dosages. In particular, no adverse effect was observed in both rats orally administered with bLf at 2 g/kg per 13 days and in humans with 1.5–9 g of rhLf per 15 days [50,51]. Non-invasive administration routes remain preferable in humans and among them, although bearing some limitations, oral management represents a safe and practical approach for Lf supplementation. Beside oral administration [48], several different other modes have been tested in animal models and humans, including aerosolized [45,46], intranasal [52], sublingual [53], intravenous [54], intraperitoneal [55], and intravaginal administration [56].

Bioavailability of orally administered Lf changes over development, with higher intestinal absorption rate in infants and lower absorption during adulthood. This is consistent with the different physiological roles of Lf during life. In fact, Lf exerts pivotal roles during lactation, both in enhancing newborn immune defense and promoting milk iron absorption. A pioneer study on intestinal iron absorption in human infants has shown that human milk helps the metal to be absorbed up to 50%, while absorption of iron from bovine milk and infant formula remains considerably lower, 5%–20% [57]. In this respect, Lf, which carries most of the iron in milk, is undoubtedly the main actor in increasing iron bioavailability from human milk [58]. In infants, gastrointestinal absorption of antigenically intact proteins is much higher due to a freely permeable gut [59]. Studies in piglets have shown that the gut is easily passed through by various macromolecules, mostly during the first few days post-partum [60,61]. Consistent with this, Lf has been shown to be efficiently transferred from the intestine into systemic circulation in a newborn [62] as well as in 3–5-month old calves [63], piglets [64], and infants [65]. In addition, the immature state of the neonatal gastrointestinal system, characterized by a moderately high pH and low secretion of proteolytic enzymes, has been correlated to the persistence of intact Lf during gastrointestinal passage [66]. So far, it is widely recognized that, in infants, Lf survives gastric digestion, thus maintaining the ability to interact with its specific receptor in the small intestine [66]. In contrast, studies on gastrointestinal digestion kinetic of Lf in adults have highlighted that rhLf from transgenic cows is completely digested [67], whereas about 60% of bLf was found undigested [68]. Therefore, in human adults, bLf appears to be more resistant than hLf against proteolytic degradation, suggesting that hLf is “designed” to be ingested orally only during infancy. In order to increase bLf bioavailability, the oral administration should be performed before meals so to avoid its gastric degradation due to very low pH during digestion.

Once in the intestine, hLf can interact with its receptor and be internalized by enterocytes, then partially vehiculated into the bloodstream and delivered to tissues. The presence of human Lf receptors (hLfRs) in the small intestine was proposed for the first time by Cox and colleagues [69]. Upon incubation of mucosal biopsies from adult human small intestine with ^59^Fe-labeled hLf, bLf, human serum transferrin (Tf), and ovo-Tf, they found that Lfs, but not serum or ovo-Tf, were able to deliver iron to human duodenal mucosa. By using the same approach, they demonstrated that iron from human Tf, but not from Lf, was taken up by reticulocytes, suggesting the peculiar role of transferrin as blood iron shuttle and ruling out the involvement of Tf in iron absorption from the intestinal mucosa [69]. It is interesting to recall that, among mammals, rat is the only species presenting Tf as the only iron-cargo protein in milk, ensuring iron absorption in rat intestine [70,71]. Therefore, rat should be considered a poor model for studying Lf intestinal absorption and tissue delivery, being devoid of Lf receptors on mucosal cells. In a subsequent study, Ashida and colleagues demonstrated that holo-hLf was internalized from the apical side but not from the basolateral one in polarized monolayers of Caco-2 cells, a human cell line of intestinal derivation, whereas holo-hTf was internalized only from the basolateral one [72].

A number of unsolved questions and controversies emerge from studies on the transepithelial transport of Lf in adults, which consists in the internalization and subsequent release of the protein in the lamina propria. Most of in vitro studies, usually performed on polarized monolayers of intestinal cell lines, have disclosed a major degradation pathway for the internalized hLf [73,74,75], and a minor one for its transepithelial transport [76], yet a very recent report showed that intact hLf and its N-lobe derivative, after cell uptake, were both released back into culture medium [77]. These contradicting data can be due to the heterogeneous features of the Caco-2 cell line as well as to different culture-related conditions [78].

On the other hand, in vivo studies on the intestinal transepithelial transport of oral administered bLf, have reported more robust evidence for the vehiculation of intact bLf into systemic circulation in adult pigs [79], rats [80], and mice [81], via both the lymphatic pathway and the portal vein [64,79,80]. Compared to a significant amount of relevant studies demonstrating Lf intestinal absorption, only a few papers have shown evidence of tissue delivery of systemic Lf. Some reports have pointed out that systemic Lf is rapidly cleared from liver after intravenous injection, both in mice and rats [82,83], thus excluding the possibility of its effective transfer to tissues. However, Fisher and coworkers demonstrated that significant levels of the exogenous glycoprotein were recorded in different organs, such as the liver, gall bladder, kidneys, spleen, and brain, in mice intra-gastrically administered with bLf, suggesting that circulating bLf is efficiently delivered to the entire body [81].

Mammalian hLfRs have been shown to reproduce the multifunctional activities of hLf, as they are differentially expressed according to tissue and cell type [66]. In fact, hLf, upon binding to its receptor, can trigger different intracellular signaling pathways [84] or undergo clathrin-mediated endocytosis and subsequent nuclear translocation [72], thus enabling Lf to act as a regulator or transregulator of cellular gene expression [16]. In 1991, Kawakami and Lonnerdal isolated the first human intestinal receptor from fetal intestinal brush-border membranes [85], while, in 2001, Suzuki and colleagues cloned and functionally studied it [86]. This receptor is currently known as intelectin-1 (ITLN-1).

ITLN1 is a high affinity Lf receptor (Kd = 10^-6^ M) able to transduce several Lf-mediated functions, ranging from facilitation of intestinal iron absorption in infants to strengthening of the immune system [87,88,89,90]. A thorough screening of adult murine tissues revealed that INTL1 is expressed in the digestive tract (esophagus, stomach, small intestine, and large intestine), nervous system (cerebellum, hypothalamus, hippocampus, pituitary gland, ganglia, and spinal cord), reproductive system (testes and ovaries) and other organs, such as the pancreas, renal cortex, lung, heart, and liver [91], thus suggesting its pivotal role into the redistribution of circulating Lf into the body. So far, in humans, INTL1 was found on the intestinal brush-border, Paneth and goblet cells [66] as well as on biliary epithelium [92].

Besides INTL1, low density lipoprotein (LDL) receptor-related protein (LRP) plays a pivotal role in Lf metabolism and signaling. It is mainly known to be involved in hepatocyte uptake of lipoproteins containing triglycerides and cholesterol through an endocytotic-mediated pathway. However, it was also shown to take part in several cellular processes, including cell migration, survival, motility, and differentiation, playing a role in different pathologies such as thrombosis, fibrinolysis, and atheroscleosis [93]. LRP is ubiquitously expressed in hepatocytes, neurons, smooth muscle cells, fibroblasts, and cholangiocytes [92], and it has been shown to be involved in the hepatic removal of bLf from plasma [94]. Moreover, bLf translocation across the blood-brain barrier (BBB) was also demonstrated to be LRP-mediated [95].

Concerning other hLfRs, CD14 was exclusively found on monocytes, asialoglycoprotein receptor (ASGPR) in liver and nucleolin in lymphocytes [66,96]. Interestingly, hLf ability to enter into the nucleus [97] is possibly mediated by both ITLN1 and surface nucleolin [98,99]. Therefore, due to the receptor specificity, hLf can exert multiple and differential functions depending on the cell system it acts upon.

Concerning bLf, its interaction with hLfRs was first described by Shin et al. [100], and its nuclear localization in human enterocytes in a further study [101], thus strengthening and partially explaining how bLf could act as a potent bioequivalent of the human homologue.

Overall, despite most of Lf-mediated molecular mechanisms still being under investigation, signal transduction following Lfs-hLfRs interaction and the ability of Lf to enter into the nucleus likely represent the main mechanisms through which Lfs exert its pleiotropic functions, including anti-inflammatory, immunomodulatory, and anti-cancer roles.

## 3. Lactoferrin and Cancer

Over the 1970s and 1980s, several different functions have been ascribed to Lf, including antibacterial, antimicrobial, and immunomodulatory abilities, yet it was only in 1994 that Bezault and coworkers presented convincing data about the anti-cancer activity of hLf in murine models of fibrosarcoma and melanoma [102]. In particular, intraperitoneal administration of hLf was found to inhibit both solid tumor growth and lung metastasis independently of the iron-saturation rate of the protein. In the same report Lf anti-cancer ability was correlated to the activation of Natural Killer (NK) cells [102]. At odds with these findings, a negative correlation between endogenous hLf expression and cancer incidence was demonstrated in different cancer cell lines, all showing a marked down-regulation of hLf transcript [103,104,105]. Lf gene silencing in cancer cells was found to be related to different molecular mechanisms, including promoter and gene hyper-methylation or direct alteration of the gene sequence [106,107,108]. Interestingly, Zhang and colleagues showed that the restoration of hLf gene expression through the use of a methyltransferase inhibitor impaired cancer cell growth and metastasis in a model of oral squamous cell carcinoma [108]. Generally, hLf and bLf have been shown to exert anti-cancer activity for both tumor prevention and treatment [18,109]. The Lf preventive effect has been demonstrated in several animal models bearing different types of tumors, including lung, tongue, esophagus, liver, and colorectal [110,111,112,113], whereas Lf treatment was found efficient in inhibiting growth, metastasis, and tumor-associated angiogenesis [18,114,115], as well as in boosting chemotherapy [116,117].

In humans, Lf application in clinical trials for cancer prevention is almost impracticable for most of the tumors, however, investigations on its potential use for the treatment of specific type of pre-cancerous lesions, in order to prevent their transformation in highly tumorigenic cells, have been carried out [118,119]. In particular, in 2002–2006, Tsuda research group investigated the inhibitory effects of orally administered bLf on the growth of pre-cancerous adenomatous colorectal polyps in a clinical trial carried out on 104 participants, with ages ranging from 40 to 75, at the National Cancer Center Hospital, Tokyo, Japan. The participants were divided into three groups, acquiring 0 (placebo group), 1.5, or 3 g of bLf daily over one year [118]. The results showed that, whereas the lower dose did not exert any significant effect, the higher dose was able to hinder the growth of colorectal polyps, in patients 63 years old or younger, when compared to the placebo group. Interestingly, serum hLf levels in patients acquiring 3 g of bLf were found to be significantly increased already after 3 months of treatment, consistent with an increase in neutrophils activity [118]. In 2014, the same group enriched the study by presenting data on the correlation between immune parameters and polyps size [119]. Trial participants with regressing polyps presented: i) increased NK cell activity and higher numbers of CD4^+^ cells in the polyps, consistent with the activation of the adaptive immunity and ii) decreased numbers of polymorphonuclear neutrophils and increased numbers of S100A8^+^ cells in the polyps, consistent with the down-regulation of inflammatory stimuli [119]. Overall, although the molecular mechanisms remain unveiled, the Tokyo clinical trial represents a pivotal upshot showing the efficiency of oral bLf administration in counteracting cancer development in humans.

Besides general clinical aspects, several molecular mechanisms underlying Lf anti-cancer ability have been unveiled, including the modulation of cell cycle, promotion of apoptosis, hindering of migration and invasiveness, as well as immunomodulation. Except for the indirect immunomodulatory pathway, the other mechanisms require the direct recognition of and selection between cancerous and normal cells by Lf, possibly involving a primary interaction with peculiar cancer cell surface receptors or a secondary one via the regulation of differential intracellular networks. To date, only few evidences have been reported for the primary recognition between Lf and cancer cell surface receptors [120,121,122]. In this respect, most of cancerous cells have a high content of proteoglycans, glycosaminoglycans (GAGs, mainly heparan and chondroitin sulfates), as well as of sialic acids, all these molecules being well-known interactors for Lfs [120,123,124]. This crude recognition could be at the base of Lf anti-cancer specificity and selectivity. The N-terminal portion of hLf, containing a unique cluster of four consecutive arginine residues (G^1^RRRR^5^), was demonstrated to be essential for hLf interaction with GAGs on the human colon carcinoma cell line HT29-18-C1 as well as on Jurkat human lymphoblastic T-cells [123,125]. Interestingly, N-terminal region of bLf, which shows a different consensus sequence (A^1^PRKN^5^), is able to interact with cell membrane-associated GAGs similarly to hLf [125]. Moreover, Riedl and colleagues have shown that phosphatidylserine, a cytoplasmic membrane component largely represented on tumor cells, is a crucial target for the specific anti-cancer activity of human Lfcin (Lfcin-H) derivatives [126]. This primary selective interaction via cell surface receptors can explain the most archaic function ascribed to Lf, i.e., its cytotoxic activity. Indeed, especially at high concentrations, both hLf and bLf and even more their derived peptides have been found to promote cytotoxicity and cell death for both prokaryotic and eukaryotic pathogens [127,128,129], as well as for cancer cells [130,131,132,133]. This activity has been mostly associated to the cationic charge of Lfs which can promote electrostatic interactions with negative charged cell surface receptors. Concerning Lf derived cationic peptides, their low specific mass weight enables them to enter and destabilize cell membrane thus easily inducing a lytic process [31]. As a matter of fact, many current treatments for cancer rely on antimicrobial peptides which display high specificity for targeting cancer cells and low toxicity for normal cells [134].

Overall, due to similar cell selectiveness, hLf, bLf, and their derived peptides have been tested and recognized to exert pivotal role in cancer prevention and treatment. Insights on the most current advances in Lf anti-cancer field are reviewed below according to the specific effects and mechanisms.

### 3.1. Lactoferrin Anti-Cancer Activity: Modulation of Cell Cycle

The mammalian cell cycle is normally tightly controlled, mainly by hormones and growth factors, and its dysregulation can lead to tumor development. Cyclins, ciclin-dependent kinases (CdKs) and their antagonists, the CdK inhibitors, are key players in the regulation of cell cycle progression. In mammals, the active complex in G1 comprises cyclin A, cyclin D–CdK4/6, and cyclin E–CdK2. The interaction between cyclin D and CdK4/6 leads to the phosphorylation of retinoblastoma (Rb) protein, which, in mid-to-late G1 phase, promotes the release of different transcription factors bound by Rb, resulting in the activation of gene transcription and DNA synthesis. The assembly and activation of cyclin E and CdK2 follows, regulating further steps required for DNA synthesis in S phase [135]. This process is tightly regulated by CdK inhibitors, which hamper the binding between the cyclin and its specific CdK. Among them, p21, p27, and p57, belonging to the CIP/KIP family, inhibit the kinase activities of cyclin D-CdK4, cyclin D-CdK6, cyclin E-CdK2, and cyclin A-CdK2.

Despite most of the chemotherapeutic agents are addressed to arrest cell cycle and to induce cytotoxicity in cancer cells, they usually do not discriminate between normal and cancer cells, thus leading to a series of adverse and dramatic effects on patients. Conversely, Lf has been described to act as a selective agent with regard to cancer and normal cells. As a matter of fact, Lf was found to mainly act as a positive regulator of proliferation in normal cells [136,137], while exerting a predominant inhibitory effect versus transformed and cancer cells [18], thus highlighting once again its innate selectivity and differential potential.

Lf is able to act as a growth factor, by enhancing, in a dose-dependent manner, DNA synthesis and cell growth of normal cells, including neonatal rat hepatocytes [138], human endometrial stroma cells [139], primary rat and human osteoblast cells [140,141], and human embryonic kidney cells [141]. Recent studies have unveiled the molecular mechanisms involved in Lf pro-proliferative effects in MC3T3-E1 osteoblast cell line in vitro [136,137]. In particular, the native forms of both bLf and hLf have been shown to shorten the cell cycle, in a dose-dependent manner, by upregulating the mRNA expression of Proliferating Cell Nuclear Antigen (PCNA), thus globally enhancing the number of cells found in S and G2/M phases [136]. Similarly, bLf significantly enhances cell growth via phosphorylation of both extracellular-signal-regulated kinase (ERK) and p38 mitogen-activated protein kinase (MAPK), while preventing the activation of c-Jun N-amino-terminal kinase (JNK) [137].

Concerning cancer cells, hLf was found to arrest cell growth, in G1 to S transition phase of the cell cycle, in a model of breast cancer, the MDA-MB-231 cells [142]. At the molecular level, hLf induced a significant decrease in the protein levels and activity of both Cdk2 and Cdk4, responsible for the activation of cyclin D and cyclin E, respectively, which, in turn, play a key role in the transition from G1 to S phase. Moreover, CdK inhibitor p21 was found increased in a p53-independent mechanism [142].

This hLf effect was also reported in four models of head and neck cancer cells, which resulted arrested in G1 to S transition upon hLf treatment [143]. Lf anti-proliferative effect was associated to the increase of CdK inhibitor p27 protein accompanied by the suppression of cyclin E. The increased levels of p27 were related to decreased phosphorylation of Akt, which determines a reduction in p27 phosphorylation and subsequent increased resistance of the protein to proteasomal degradation [143].

In the study by Zhang et al. [144], the inhibitory effects of bLf on the growth of four breast cancer cell lines (T-47D, MDA-MB-231, Hs578T, and MCF-7) but not on the normal breast cell line MCF-10-2A, were reported, thus confirming the selectivity of Lf. Interestingly, only the full-length glycoprotein, especially in its iron-saturated form, but not its derived peptides, was active in this inhibitory effect. The Authors showed that bLf-mediated arrest of cell cycle was associated to the increase of phospho-AMP activated serine/threonine protein kinase (AMPKα) and decreased levels of both phosphorylated and total forms of mTOR, the nutrient/energy sensor kinase involved in several cell pathways including cell survival. Therefore, the downregulation of total and phospho-mTOR following the activation of AMPKα signaling was proposed as the molecular mechanism responsible for bLf-mediated cell cycle arrest [144].

In line with these studies, Chea et al. [145] showed that bLf was able to induce cell cycle arrest also in a model of oral squamous cell carcinoma by increasing levels of both phospho-p53 and CdK inhibitor p21 and decreasing the expression of cyclin D1, a regulator protein required for progression through G1 phase of cell cycle. Importantly, bLf neither down-regulated phospho-p53 and cyclin D1 nor up-regulated p21 levels in RT7 cells, a normal human oral keratinocytes line [145].

Lastly, also bovine Lfcin (Lfcin-B) was described to be able to block cell cycle in human colon cancer CaCo-2 cells [146]. Indeed, Lfcin-B was able to induce a slight but significant prolongation of S phase when compared to untreated cells by down-regulating the level of cyclin E1 [146].

### 3.2. Lactoferrin Anti-Cancer Activity: Induction of Apoptosis

The term ‘apoptosis’ describes the biological mechanism by which a cell actively pursues a path toward its death upon specific stimuli. As a highly selective process, apoptosis is pivotal in both physiological and pathological conditions. Principal mechanisms described for the induction of apoptotic process include the extrinsic and the intrinsic pathways. The extrinsic one, also named ‘the death receptor pathway’, requires a ligand to bind to a death receptor. Among them, the type 1 tumor necrosis factor (TNF) receptor (TNFR1) and the related protein Fas, as well as their ligands, TNF and Fas ligand (FasL) respectively, are the most studied [147]. Along with the binding of the death ligand to the death receptor, an adaptor protein intervenes forming a protein complex known as the death-inducing signaling complex (DISC) [148]. DISC then initiates the assembly and activation of pro-caspase 8, which in turn activates caspase-3 or cleaves Bid, a family member of Bcl-2, leading to the formation of the apoptosome and to final activation of caspase-9 [147]. In the intrinsic pathway, internal stimuli, such as DNA damage, severe oxidative stress and hypoxia, are responsible for the induction of apoptosis. It involves the release of pro-apoptotic molecules such as cytochrome-c from the mitochondria to the cytoplasm, with the downstream activation of proteins belonging to the Bcl-2 family [149]. These latter group of regulatory proteins include both pro-apoptotic (e.g., Bax, Bak, Bad, Bcl-Xs, Bid, Bik, Bim, and Hrk) and the anti-apoptotic (e.g., Bcl-2, Bcl-XL, Bcl-W, Bfl-1, and Mcl-1) proteins, whose ratio directly determines the cell fate [150]. Furthermore, all these regulators of apoptosis are modulated by several upstream intracellular pathways, including Akt, ERK1/2, and JNK.

In cancer, the succession of genetic changes accumulating in transforming cells results, besides higher proliferation rate and migration/invasion features, in the cell ability to evade death-inducing signaling. Therefore, reduced apoptosis or cell resistance play a pivotal role in the carcinogenesis. In general, cancer cells escape apoptosis by disrupting the balance between pro-apoptotic and anti-apoptotic proteins, reducing caspase activity or affecting death receptor signaling. In this scenario, new drugs or treatment strategies that can rebalance the proper apoptotic response for cancer cells can potentially function as chemotherapy.

Among its multi-faceted activities, Lf has also been described to be a potent activator of apoptosis in many different type of cancers [18,151]. HLf-induced apoptosis in Jurkat leukemia T cells has been described to occur in a dose-dependent manner via the activation of JNK and caspase 9 and 3 which in turn resulted in increased levels of Bcl-2 phosphorylation [152]. By contrast, upon treatment with a JNK inhibitor, hLf challenging no longer induced any apoptotic process in the same cell line [152]. A deeper analysis of the Lf-induced Bcl-2-dependent apoptotic signaling showed that hLf reduces the formation of the Rb/E2F1 complex by inducing the phosphorylation of Rb, thus promoting the release of E2F1 transcription factor, a downstream regulator for cell proliferation and apoptosis via activation of its target genes [153].

By injecting an adenovirus expressing the hLf cDNA into the tumor site in mice bearing EMT6 breast cancer, Wang and coworkers demonstrated that hLf could significantly reduce tumor growth. At the molecular level, hLf was found to induce the apoptotic process by decreasing the anti-apoptotic Bcl-2 and increasing the pro-apoptotic Bax and caspase 3 expressions [154]. Moreover, rhLf produced in the yeast Pichia pastoris has been shown to induce apoptosis through plasma membrane blebbing, cell shrinkage, and chromatin condensation, as well as the disruption of F-actin cytoskeleton organization in the human breast cancer cell line MDA-MB-231 [155].

Moreover, bLf was described to induce apoptosis in stomach cancer cell line SGC-7901 by down-regulating Akt intracellular signaling [156]. In particular, bLf treatment induced the dephosphorylation of Akt in Thr308 and Ser473, thus blocking downstream apoptotic regulators [156].

BLf was also demonstrated to induce apoptotic extrinsic pathway by up-regulating Fas signaling in the colon mucosa of azoxymethane-treated rats [157]. In particular, upon bLf treatment Fas protein, but not TNF-R1vexpression, was up-regulated with respect to the vehicle group, along with an increase in both caspase-8 and caspase-3 levels. Furthermore, immunohistochemistry revealed Fas-positive cells and apoptotic cells especially within the proximal colon region. Notably, the incidence of tumors inversely correlated with the areas positive to Fas and apoptosis [157]. In another study, the same group showed that, altogether with the induction of Fas, also two pro-apoptotic members of the Blc-2 family, Bid and Bax, resulted increased in tumor sites in rat fed with bLf [158].

In a recent paper, Jiang and Lonnerdal described bLf and Lfcin-B, the latter in both linear and cyclic forms, as potent pro-apoptotic agents against the colorectal cancer cell line HT-29, by inducing different intracellular pathways [131]. In particular, transcriptome analysis and immunoblotting allowed to reveal that levels of several intracellular mediators involved in cell cycle arrest and apoptosis were increased by treatment with either linear or cyclic Lfcin-B, suggesting that the glycoprotein and Lfcin-B can exert their anti-cancer activity by triggering different pathways [131]. It is relevant to note that no cytotoxic and pro-apoptotic effects were recorded on normal human intestinal epithelial cells, once again confirming the high selectivity of Lf and its derivate peptides.

BLf, both in its apo- and holo forms, was found to inhibit expression of survivin, a 16 kDa protein overexpressed in several cancer cells [159]. Survivin is able to bind to pro-apoptotic molecules, including caspases, thus inducing a blockade of apoptotic process [160]. The study by Gibbon and colleagues reported that both apo- and holo-bLf significantly induced apoptosis in two breast cancer cell lines, MDA-MB-231 and MCF-7, by decreasing Bcl-2 and pro-caspase-3 expressions, along with a significant reduction in survivin levels. Importantly, no cytotoxic and pro-apoptotic affect was detected in breast normal MCF-10-2A cell line [159]. Moreover, the authors underlined the great potentiality of bLf as inhibitor of survivin since, to date, no survivin inhibitors have passed clinical trials due to their high toxicity [161].

Recently, the vacuolar H^+^-ATPase (V-H^+^-ATPase) was described as a potential target for bLf in highly metastatic breast cancer cell lines [121,122]. BLf was found to induce apoptosis, along with a significant decrease in V-H^+^-ATPase-mediated extracellular acidification rate, in two highly metastatic breast cancer cell lines, Hs 578T and MDA-MB-231, displaying a prominent localization of V-H^+^-ATPase at the plasma membrane, but not in the lowly metastatic T-47D or in the non-tumorigenic MCF-10-2A cell lines [121]. By a similar approach, bLf was described to selectively act as pro-apoptotic agent on highly metastatic prostate PC-3 and osteosarcoma MG-63 cell lines, both displaying aberrant plasma membrane localization of V-H^+^-ATPase [122]. Again, non-tumorigenic BJ-5ta cell line did not show sensitivity to bLf treatment. Overall, these studies suggest a common mechanism of action of bLf against highly metastatic cancer cells exhibiting plasmalemmal V-H^+^-ATPase, a promising perspective for its application in the treatment of these cancers.

Finally, Lfcin-B was also described to be involved in the induction of apoptosis [162,163,164]. Lfcin-B was found to promote reactive oxygen species (ROS)-dependent induction of apoptotic process in a human leukemic cell line [162], whereas its nine amino acids variant was demonstrated to activate apoptosis by up-regulating caspases 3 and 9 via ROS induction in a human ovarian cancer cell SK-OV-3up [164]. Moreover, pepsin-digested-Lf peptides exerted pro-apoptotic effect in a human oral squamous cell carcinoma cell line by promoting the cleavage of caspase-3 and poly (ADP-ribose) polymerase (PARP) as well as by inducing the phosphorylation of ERK1/2 and JNK [165].

Lfcin-B was also shown to significantly inhibit proliferation of non-small cell lung cancer NCI-H460 (H460) cells in vitro by promoting apoptosis through the stimulation of caspase-3 and caspase-9 and by preventing survivin expression [166]. Moreover, vascular endothelial growth factor (VEGF) was down-regulated at both transcriptional and translational level along with an increase of intracellular ROS production by inhibition of antioxidant enzymes. Of note, upon Lfcin-B treatment tumor growth in the H460-bearing mice model was also prevented [166].

### 3.3. Lactoferrin Anti-Cancer Activity: Inhibition of Cell Migration, Invasion, and Metastasis

Beside increased proliferation rate and resistance to apoptosis, cancer cells acquire migratory and invasive phenotypes which allow them to detach from the primary site and enter the circulatory system, thus metastasizing into distant sites. Cell migratory activity is a fundamental process during essential physiological processes including morphogenesis, wound healing and tissue regeneration. This type of migration, known as ‘collective cell migration’, involves large sheets of cells, rather than single cell, with tight intercellular connections. In adults, wound healing and tissue regeneration are the predominant processes where physiological cell migration occurs. Indeed, any break in the skin has to be rapidly repaired to avoid blood loss and the risk of infection. Importantly, inflammatory mediators are usually involved in wound healing, thus promoting the regeneration of dermal and epidermal structures. However, chronic inflammatory processes can be detrimental to such healing, leading to non-repairing wounds.

Interestingly, bLf was found to directly stimulate the proliferation and migration of fibroblasts, predominant cells in the dermis responsible for the generation of granulation tissue in wound healing, and keratinocytes, essential cells for re-epithelialization [167,168]. Moreover, addition of bLf to differentiating HaCaT human keratinocytes led to increased transepithelial electrical resistance (TER), a marker of epithelial barrier function as well as to upregulation of two differentiation markers, involucrin and filaggrin [169]. BLf was also described to promote hyaluronan synthesis in normal human dermal fibroblasts by increasing hyaluronan synthase 2 [170], suggesting that Lf-stimulated wound healing could be related to up-regulation of hyaluronan. In corneal epithelial wound healing, bLf promotes wound closure in human corneal epithelial cells [171].

The rhLf was shown to stimulate re-epithelialization of porcine second-degree burns [172], and bLf favored healing of corneal alkali wounds in BALB/c mice, associated with down-regulation of interleukin (IL)-1α and β [173]. Of note, bLf was demonstrated to promote rapid wound healing in the post-surgical treatment of patients suffering from bisphosphonate-related osteonecrosis of the jaws [174].

As expected, Lf showed selectivity by efficiently inhibiting migration and/or invasion in different types of cancer cell models [36,159,175,176]. Despite most of the studies do not detail the molecular mechanisms at the base of Lf anti-migratory and anti-invasive activities, two recent investigations have shed some light on bLf ability to revert epithelial-to-mesenchymal transition (EMT) process in both oral squamous cell carcinoma (OSCC) [176] and glioblastoma [36] cell lines. EMT is recognized as one of the main processes involved in cancer metastasis, where cells that gain a more invasive phenotype become able to infiltrate blood vessels as well as surrounding and distant tissues [177]. Different transcriptional factors play a crucial role, including Signal Transducer and Activator of Transcription 3 (STAT3) [178], Snail, and Twist [179]. Indeed, Snail and Twist, overexpressed in highly metastatic malignant tumors, repress cell-to-cell adhesion molecules, such as cadherins. On the other hand, such tumors show high levels of vimentin, a major intermediate filament protein, which has been positively associated with cancer aggressiveness, progression, and poor outcomes [180].

Chea et al. [176] showed that bLf was efficient in counteracting invasiveness of the OSCC line, HOC313, by reverting EMT process. At the molecular level, upon binding to LRP1, bLf suppressed Twist expression by downregulating ERK1/2 phosphorylation, along with an up-expression of E-cadherin and the decrease of vimentin levels. Additionally, the in vitro results were confirmed also in xenografts of mice orally administered with bLf, showing less cancer cell infiltration and increased levels of E-cadherin [176].

**Figure 2:**
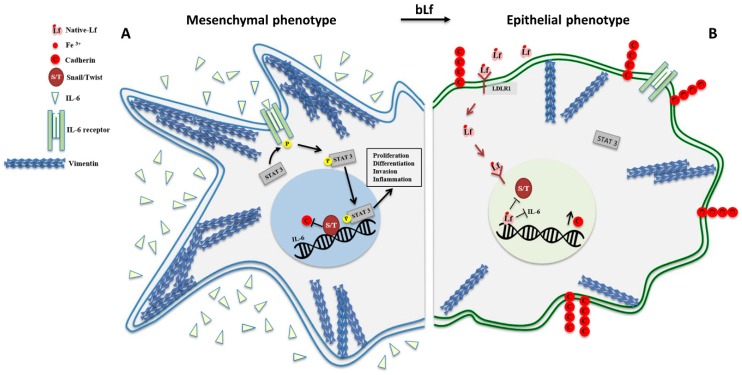
Schematic representation of bovine Lactoferrin (bLf) efficiency in counteracting migration and invasion by reverting epithelial-to-mesenchymal transition (EMT) process in cancer cells. (**A**) Mesenchymal phenotype, characterized by a spindle-like shape, presents high levels of vimentin and Snail/Twist (S/T), down-regulation of cadherins’ expression and p-STAT3-activation by interleukin (IL)-6. All these factors let the cell to acquire an invasive phenotype able to detach from the primary site and invade surrounding tissues and blood vessels. (**B**) Epithelial phenotype upon bLf treatment, which is able to revert EMT by its multi-targeting activity. After LRP1-mediated uptake and nuclear localization, bLf down-regulates vimentin, Snail and Twist expression, thus increasing cadherins’ levels. Moreover, bLf inhibits IL-6-mediated STAT3 activation.

In our study, native and holo-bLf were able to partially or completely hinder cell migration in a human glioblastoma cell line, the GL-15. In particular, depending on its iron saturation rate, bLf was efficient in down-regulating both vimentin and Snail expression, while inducing a notable increase in total cadherins’ level. Moreover, IL-6/STAT3 axis, another pivotal pathway associated to glioblastoma cell migration ability, was found to be strongly inhibited upon bLf treatment, with the holo-form more efficient than the native counterpart. Interestingly, the differential efficiency was associated to higher accumulation rates of holo-bLf in both cell cytoplasm and nucleus with respect to the native form [36]. A schematic representation of molecular mechanisms targeted by bLf for EMT reverting is shown in Figure 2. 

Along with anti-migration and anti-invasion effects, Lf was primarily reported to affect cancer metastasis. As described above, in 1994 Bazault and colleagues reported the first evidence for the anti-metastatic effect of Lf in rat models [102]. Since then, several studies have reported the Lf anti-metastatic ability against other types of cancers [87,88,181,182]. In particular, subcutaneous administration of apo-bLf and Lfcin-B in mice inoculated with two different cancer cell lines, B16-BL6 melanoma and L5178Y-ML25 lymphoma cells, resulted in significant inhibition of liver and spleen metastasis for L5178Y-ML25 cells and lung metastasis for B16-BL6 cells, as well as of tumor-induced blood vessel formation [181]. Moreover, apo-bLf was significantly efficient in suppressing the growth of B16-BL6 cells throughout the whole experimental period (21 days), whereas Lfcin-B had an effect only during the first 8 days [181]. Interestingly, neither apo-hLf nor holo-bLf were able to exert the same functions, suggesting species and iron-saturation specificity for this activity.

The oral administration of bLf and Lfcin-B to BALB/c mice bearing subcutaneous implants of the highly metastatic colon carcinoma, 26 reduced lung metastatic colony formation [87]. While Lfcin-B was by itself active in this inhibitory effect, bLf showed a significant activity depending on the activation of white blood cells towards asialoGM1^+^ and/or CD8^+^ phenotypes [87].

Recently, Wei and colleagues demonstrated that Lf deficiency enhanced melanoma metastasizing to lungs in an Lf KO mouse model by recruiting myeloid derived suppressor cells (MDSCs). Molecular studies showed that TLR9 signaling, negatively involved in MDSC-mediated cancer metastasis, was highly repressed and that exogenous Lf administration was able to revert the phenotype by enhancing TLR9 pathway as well as by inducing MDSCs differentiation and apoptosis [182].

### 3.4. Lactoferrin Anti-Cancer Activity: Immunomodulation

To date, it is well established that the tumor microenvironment, mainly composed and coordinated by inflammatory cells, is a crucial component in the tumor progression or inhibition. The inflammatory participants to cancer microenvironment principally involve leukocytes, including dendritic cells, neutrophils, and macrophages, as well as lymphocytes, all able to secrete various inflammatory mediators, such as cytokines, cytotoxic molecules, including ROS and soluble mediators of cell killing, such as TNF-α and interferons (IFNs) [183]. The fine interplay between cancer and immune cells can decide the tumor fate. Several studies have highlighted the pro-cancerogenic role of tumor-infiltrated leukocytes through release of some cytokines, such as IL-6 and IL-1β, found to be increased in different cancer models [184,185]. On the other hand, some subclasses of leukocytes, such as NK cells and cytotoxic T cells, display a potent antitumor activity [185]. Therefore, the immune response can exert both tumorigenic and anti-tumorigenic activities depending on the balance of innate or adaptive components and on the subtype of cancer it is connected to. In general, a well-orchestrated acquired immune response has to be considered as anti-tumorigenic, whereas chronic inflammation induced by unbalanced innate or acquired immune response is likely pro-tumorigenic. In this scenario, molecules able to boost cytotoxic immune components as well as to shut down pro-tumorigenic inflammatory mediators could be considered potentially good candidates as adjuvant to standard anti-cancer therapies. In this regard, Lf has proven to potentiate adaptive immune response as well as to act as a potent anti-inflammatory agent [17,102,186]. Even if the molecular mechanism is still under debate, the findings that both hLf and bLf are able to enter inside host nucleus [97,101] and to bind human DNA have shed some light on the possible mechanism by which the glycoprotein can modulate gene expression, thus exerting its anti-inflammatory activity [17].

HLf was reported to significantly increase NK cells-mediated cytotoxicity against breast and colon cancer cell lines [120]. Interestingly, hLf pre-treatment of NK cells or target cells enhances NK cytotoxicity activity and target cell sensitivity to lysis, respectively. Consistently, in a murine model of cervical carcinoma expressing hLf, Shi and Li demonstrated that the reduction of tumor growth in the hLf-treated group was associated to an induction of NK cells activity and an increase of CD4^+^ and CD8^+^ T lymphocytes in peripheral blood. Moreover, hLf-treated mice showed an increase of serum INF-γ, IL-2, and TNF-α, along with a down-regulation of serum IL-4 and cancer tissue VEGF [187].

In in vitro and in vivo models of head and neck squamous cell carcinoma, rhLf was demonstrated to induce a dose-dependent growth inhibitory effect by modulating inflammatory and immune response [188]. The in vitro rhLf-mediated inhibition of cancer proliferation was associated to up-regulation of NF-kβ signaling as well as to the down-regulation of pro-inflammatory and pro-metastatic cytokines, including IL-8, IL-6, granulocyte macrophage colony-stimulating factor and TNF-α. Moreover, in tumor-bearing mice the oral administration of rhLf resulted in a 20-fold increase of lymphocytes and 75% tumor growth inhibition when compared with vehicle group. Of note, in mice depleted of CD3^+^ cells all rhLf treatment was no longer efficient [188].

The oral administration of bLf was described to have a potent anti-carcinogenic activity by increasing the expression of mucosal IL-18 mRNA in the small intestine of mice [189]. The same effect, though at different extent, was promoted by the bLf pepsin hydrolysate and Lfcin-B, both showing a positive influence on IL-18 mRNA expression in the murine small intestine and organ culture, respectively. Moreover, both bLf and Lfcin-B, in addition to IL-18 activation, were able to induce a significant up-regulation in caspase-1 activity in peritoneal macrophages, which is directly involved in the cleavage of pre-mature to mature form of IL-18 protein [186]. Importantly, this bLf-mediated activation of IL-18 by caspase-1 was found to be dependent on IFN-γ expression by peritoneal macrophages. As a matter of fact, IFN-γ KO mice were insensitive to bLf oral administration resulting in unchanged levels of active caspase-1 and mature IL-18. On the other hand, bLf was still efficient in inhibiting tumor growth in IFN-γ KO mice by activating IFN-α/IL-7 pathway, thus strengthening the concept that bLf can act as a multi-targeting agent [189].

BLf was reported to inhibit tumor growth in in vitro experiments on a human lung cancer cell line, A549, as well as in a murine model of lung cancer overexpressing VEGF. Results showed that bLf treatment reduced VEGF expression in a dose dependent manner, along with a significant reduction of global cytokine levels, including the pro-inflammatory TNF-α and IL-6 and the anti-inflammatory IL-4 and IL-10 [190]. Additionally, bLf hindered the proliferation, migration, and bone resorption in an in vitro model of osteosarcoma by attenuating inflammatory processes through the reduction of IL-1β, IL-6, RANKL, and phospho-NF-kB [191].

Other than the direct modulation of immune response, Lf strategically acts as a potent anti-inflammatory agent by scavenging ROS, pro-oxidant agents able to both promote DNA damage and induce/sustain inflammatory disorders, drastically contributing to cancer development. Lf is able to maintain the physiological balance of ROS levels by either direct binding of free iron, one of the principal actors involved in ROS production, and regulation of key antioxidant enzymes, thus globally protecting host from ROS-mediated cell and tissue damages [192]. In this respect, a recent study by Mohammed and colleagues have shown that bLf significantly down-regulated the activity of liver antioxidant enzymes, namely catalase, glutathione peroxidase, and superoxide dismutase, along with an increase of the concentration of hepatic reduced glutathione, in a murine model of diethylnitrosamine-induced hepatocarcinogenesis. Accordingly, bLf treatment promoted the decrease of serum inflammatory markers and significant amelioration in hepatic histological structures [193].

Finally, bLf has been recently proven to protect against iron disorders by modulating immune response and down-regulating pro-inflammatory cytokines, such as IL-6, in both in vitro [20,194,195] and in vivo [46] models as well as in clinical trials [48].

### 3.5. Lactoferrin as a Cargo for Cancer Targeting

In cancer treatment and therapy, different chemical and biological compounds have been tested in order to increase specificity for tumor cells, without affecting the quality of life of patients. Indeed, although all tested molecules are endowed with great anti-cancer potential, most of them lack selectivity, resulting in high cytotoxicity for non-cancer tissues [196]. Hence, specific targeting is becoming an imperative feature of new compounds in cancer treatments. In this regard, the development of engineered nanoparticles (NPs), able to act as a specific carrier, is greatly improving the possibility to specifically deliver drugs. NPs-mediated cancer targeting is ensured by passive and active processes which can drive NPs efficient localization in the tumor microenvironment and promote their uptake by cancer cells, respectively. The specific cell uptake is usually guaranteed by the presence of ligands able to interact with surface membrane receptors overexpressed on target cells. The use of nanocarriers presents several advantages other than selectivity, including long-term systemic longevity and easy permeation into cellular membranes. Moreover, nanoparticles-mediated targeting has been proven to decrease multidrug resistance [197,198]. To date, several ligands have been tested for efficient targeting, such as organic molecules (e.g., folic acid), antibodies or their fragments, peptides and whole proteins, including Lf [199].

The simple conjugation of apo- or holo-bLf with Doxorubicin (Dox) was demonstrated to improve internalization and nuclear retention of Dox into prostate cancer cells, along with a four-fold increase of Dox-mediated cytotoxicity [200]. Moreover, the complex, but not Dox alone, was efficient in overcoming multi-drug resistance in an advanced drug resistant cancer cell line. Finally, mice orally administered with holo-bLf-Dox conjugates showed better survival rates, a marked reduction in tumor growth and Dox-mediated general toxicity, neurotoxicity and cardiotoxicity, as well as an increase in serum levels of TNF-α, IFN-γ, CCL4, and CCL17 [200].

The use of Lf-loaded liposomes as anti-cancer drug carrier has also been tested [201,202]. In particular, bLf-containing polyethylene glycol (PEG)-modified liposomes have been employed in the targeting of Dox in in vitro and in vivo models of hepatocarcinoma, showing a relevant effect in improving Dox cellular uptake, which was associated to the presence of asialoglycoprotein receptors on cancer cell membrane, and to a significant inhibition of tumor growth in mice bearing HepG2 xenografts compared to mice injected intravenously with the sole Dox-loaded PEGylated liposomes [201]. Moreover, the intravenous injection of Dox-loaded holo-bLf-liposomes in a murine model of breast cancer resulted, along with a high accumulation of the nanocomposites in the tumor site, in tumor suppression and in a higher induction, when compared to mice treated with the sole Dox-loaded liposomes, of hydrogen peroxide conversion to oxygen, due to the release of ferric ions following holo-bLf degradation [202]. Similarly, bLf-containing micelles were used for the efficient targeting of both rapamycin and wogonin to treat breast cancer cells [203]. In general, bLf-micelles showed good serum stability and blood compatibility. Additionally, they were found to increase cytotoxicity of rapamycin and wogonin in terms of tumor inhibition in in vitro and in vivo models as a result of enhanced active targeting [203].

Concerning NPs, different anti-cancer approaches based on Lf-mediated targeting have been reported [54,204,205]. In particular, Lf was employed as carrier for NPs loaded with anti-cancer drugs or DNA encoding for anti-cancer effectors. 5-Fluorouracil (5-FU), entrapped in bLf-NPs with great efficiency and high storage stability, showed enhanced receptor-mediated uptake, prolonged intracellular retention and higher cytotoxicity in a melanoma cell line, when compared to free 5-FU [204]. Moreover, bLf and Lfcin-B-bearing dendriplexes containing DNA encoding for TNF-α were applied in the treatment of mice implanted with both melanoma and carcinoma cells [54]. The intravenous injection of dendriplexes resulted in the specific expression of TNF-α in tumor tissue with very low levels of non-specific gene expression in the liver. In one month, the suppression of 60% of carcinoma tumors and up to 50% of melanoma tumors was observed, with no side effects for animals [54]. Similarly, the same group demonstrated that the system was highly safe and selective in the suppression of prostate cancer cells in mice [205].

Importantly, Lf was found efficient in the specific targeting of central nervous system (CNS) by overcoming blood–brain barrier (BBB) [206,207,208,209]. As a matter of fact, one of the most critical limitation for the treatment of CNS pathologies, including cancer, is represented by BBB which restricts the delivery of therapeutic agents to the brain. The use of Lf as carrier for CNS treatment is due to the high expression of LRP1 on cells constituting BBB and even more on glioma cells, the most recurrent type of cancer in the brain [66,210]. Overall, studies employing Lf-bearing NPs co-loaded with different type of chemotherapeutics in the treatment of glioblastoma reported a good safety profile, an enhanced permeation of BBB and efficient drug delivery to glioma cells [206,207,208,209].

Taking all these evidences together, it is clear that the use of Lf as a cargo molecule for cancer therapy is sustained by several advantages: i) high stability and resistance to proteolysis which makes it suitable for NPs preparation and long-term storage; ii) null or low antigenicity when intravenously infused; iii) great selectivity for cancer cells due to the overexpression of its target receptors on their plasma membrane; iv) anti-cancer properties which can markedly improve chemotherapy efficiency; and v) proven safety even at high dosage.

## 4. Conclusions

None of the current cancer treatments, including radiotherapy, chemotherapy, immunotherapy, and surgery, although beneficial, represent a full ideal approach, as all more or less affect the quality of life of the patients. Recently, the use of nutraceuticals as natural compounds corroborating standard therapy is emerging as a promising tool for their relative abundance, bioavailability, safety, low-cost effectiveness, and immuno-compatibility with the host. Moreover, the oral route makes them very easy to be administered and well tolerated. However, to date, most of nutraceuticals applied in cancer prevention and treatment have shown limited effectiveness and even, in some cases, antagonist activity against standard therapies. In this context, a molecule able to both prevent and treat cancer as well as to boost conventional clinical approaches has to be considered a powerful weapon to unsheathe.

As emerging from the data presented in this review, Lactoferrin, and especially its bovine milk derivative form, is potentially a strong candidate for anti-cancer alternative treatment with the same advantages but almost null side effects characterizing standard therapies. Indeed, our feeling is that Lf will find productive applications in synergy with more conventional therapies, most likely allowing these latters to be administered at lower, more sustainable doses. As a matter of fact, Lf shows high bioavailability after oral administration, high selectivity toward cancer cells, no significant side effect and a wide range of molecular targets controlling tumor proliferation, survival, migration, invasion, and metastasization. Of note, Lf, according to the system it acts upon, is able to trigger differential outcomes. Indeed, Lf exerts positive or negative effects on cell cycle progression and cell migration towards normal and cancer cells, respectively. Moreover, Lf can prevent development or inhibit cancer growth by boosting adaptive immune response. The ability of Lf to cross the blood–brain barrier makes it a powerful tool to treat brain tumors. Lastly, Lf was recently found to be an ideal carrier for chemotherapeutics, thus globally appearing as a promising tool for cancer prevention and treatment, especially in combination therapies.
